# Editorial: Global excellence in implementation science: Europe

**DOI:** 10.3389/frhs.2024.1441375

**Published:** 2024-07-08

**Authors:** Ana Gama, Tayana Soukup, Sónia Dias

**Affiliations:** ^1^ NOVA National School of Public Health, Public Health Research Centre, Comprehensive Health Research Center (CHRC), REAL, NOVA University Lisbon, Lisboa, Portugal; ^2^ Department of Surgery and Cancer, Faculty of Medicine, Imperial College London, London, United Kingdom; ^3^ Health Service and Population Research Department, Centre for Implementation Science, King's College London, London, United Kingdom

**Keywords:** health interventions, implementation science, complex interventions, evaluation research, implementation research

**Editorial on the Research Topic** Global excellence in implementation science: Europe

Major public health concerns worldwide derive from emerging societal challenges, such as for example, the aging of the population, the increase in chronic diseases (largely associated with lifestyle risk factors), multimorbidity, low healthy life expectancy, mental health problems, loneliness, social and health inequities (1, 2). These challenges result in increased demands to and growing pressure on the healthcare systems to be responsive and effectively address populations' health needs, providing quality care while ensuring sustainability (1, 2). Although many countries in Europe offer universal health coverage (defined by the WHO as everyone having access to effective, good-quality health services when they need them) no matter their financial status, many people are still out of the scope of health systems and have unmet health care needs (3).

Innovative evidence-based interventions in health promotion, prevention, treatment, rehabilitation, and palliative care can address health needs and improve the health and wellbeing of individuals and communities. It is widely acknowledged that health interventions embed aspects of complexity that need to be considered in its design and development (4). Complex health interventions are usually described as interventions that comprise several interacting components, target diverse groups or organisational levels, aim to reach a variability of outcomes, and involve a high degree of flexibility or tailoring (5). However, the contexts in which complex interventions are implemented are also complex (4). Health interventions are implemented across varied health systems and contexts where multiple interrelated factors influence health and broader societal outcomes at the individual, population and system levels (4). In this sense, an innovation successfully implemented in one setting, does not necessarily operate the same way in another setting. Evidence on what, for whom, in what settings, and under what conditions an intervention works is key to building on findings across studies and contexts and informing researchers, practitioners, and policy makers' practice.

This collection titled “*Global excellence in implementation science: Europe*” features a range of recent health interventions that shed light on the progress made across the breadth of the Implementation Science and reflect on the future challenges faced by researchers across borders. Each article contributes to progressing evidence in the field by addressing public health concerns affecting different population groups through scientifically sound research protocols. Collectively, these articles reflect the methodological diversity within the implementation research, ranging from a cross-sectional survey, a scoping review, a qualitative study, and a realist evaluation using a mixed-methods approach, while also highlighting the challenges in advancing the field.

The first paper of this collection, “*Applicability of exercise and education programmes for knee osteoarthritis management to Switzerland*”, by Ettlin et al., assesses the applicability of six exercise and education programmes approved by the Osteoarthritis Research Society International for the conservative management of knee osteoarthritis to the Swiss health care system. Through a cross-sectional survey study using the RE-AIM framework, the authors found that four programmes scored higher than 7 and included supervised exercise sessions and education with the goal that the participants understand the diagnosis and the management of the disease, while the two lower rated programmes focused on exercise counselling or weight reduction. In this paper, the authors highlight the need of implementing a renowned and established programme to contribute to closing the existing evidence-performance gap in clinical practise. Given the high prevalence of knee osteoarthritis in Switzerland and the lack of knowledge of the beneficial effects of exercise and education, the authors argue that structured and systematic programmes such as GLA:D® would be the most applicable to the Swiss health care system, suggesting that it could be implemented successfully in the country with only few adaptations.

In the second paper, “*Contextual determinants influencing the implementation of fall prevention in the community: a scoping review*”, van Scherpenseel et al. draw from qualitative content analysis of the literature and four consensus meetings with health and social care professionals in Utrecht, the Netherlands, to identify barriers and facilitators of the implementation of fall prevention interventions within the CFIR domains. The authors highlight that multiple contextual determinants to implementation are reported, emphasizing that successful implementation of fall prevention interventions in the community is challenging since there is not one single factor that can be identified as a key barrier or facilitator. Some determinants are considered important to address, regardless of the context where the implementation occurs, and include accounting for time constraints and financial limitations, considering the needs of older adults, and assuring broad cross-sector collaboration and coordination in multifactorial interventions.

The third paper, by Dannapfel et al., is titled “*Implementing smoking cessation in routine primary care—a qualitative study*”. Through in-depth interviews with health care professionals working in primary care in Sweden, the authors explore professionals' perceptions about smoking cessation practice in routine primary care and the use of digital tools in this work. While poor continuity of staff and negative attitudes towards preventative work are reported as challenges to smoking cessation practice, societal changes towards increased awareness of the health risks of tobacco use and shifting social norms regarding the acceptance of smoking are perceived as contributing factors to a normalization of speaking about smoking in primary care practice.

The final paper of this collection, by Fredsted Villadsen et al., is titled “*Unlocking the mechanisms of change in the MAMAACT intervention to reduce ethnic disparity in stillbirth and newborns*’ *health: integration of evaluation findings*”*.* It focuses on a complex intervention implemented in Denmark, consisted of training of antenatal care midwives in cultural competencies and intercultural communication combined with health education materials for the expecting parents about symptoms of pregnancy complications. After a realist evaluation through a qualitative in-depth implementation analysis and a process evaluation embedded in a cluster randomized trial, this article integrates the MAMAACT evaluation results to identify how the activities were affected by contextual enablers and barriers to produce mechanisms of change. The article also analyses the adaptions needed in future interventions to improve pregnancy outcomes of women with immigrant backgrounds in Europe.

In conclusion, the diverse and complex nature of health interventions requires a nuanced understanding of the contextual factors that influence their implementation and effectiveness. The studies in this collection highlight the importance of adaptability, stakeholder engagement, and the consideration of multiple determinants to successfully implement and scale interventions across different settings. As implementation science continues to evolve, it must prioritize the development of tailored strategies that address specific local needs while building on robust evidence from diverse contexts. This approach will not only enhance the effectiveness of health interventions but also contribute to the overarching goal of equitable and sustainable healthcare systems worldwide.
